# Demographic and Clinical Overview of Hospitalized COVID-19 Patients during the First 17 Months of the Pandemic in Poland

**DOI:** 10.3390/jcm11010117

**Published:** 2021-12-26

**Authors:** Robert Flisiak, Piotr Rzymski, Dorota Zarębska-Michaluk, Magdalena Rogalska, Marta Rorat, Piotr Czupryna, Beata Lorenc, Przemysław Ciechanowski, Dorota Kozielewicz, Anna Piekarska, Maria Pokorska-Śpiewak, Katarzyna Sikorska, Magdalena Tudrujek, Beata Bolewska, Grzegorz Angielski, Justyna Kowalska, Regina Podlasin, Włodzimierz Mazur, Barbara Oczko-Grzesik, Izabela Zaleska, Aleksandra Szymczak, Paulina Frańczak-Chmura, Małgorzata Sobolewska-Pilarczyk, Krzysztof Kłos, Magdalena Figlerowicz, Piotr Leszczyński, Izabela Kucharek, Hubert Grabowski

**Affiliations:** 1 Department of Infectious Diseases and Hepatology, Medical University of Białystok, 15-089 Bialystok, Poland; pmagdar@gmail.com; 2 Department of Environmental Medicine, Poznan University of Medical Sciences, 60-806 Poznan, Poland; 3 Integrated Science Association (ISA), Universal Scientific Education and Research Network (USERN), 60-806 Poznan, Poland; 4 Department of Infectious Diseases, Jan Kochanowski University, 25-369 Kielce, Poland; 5 Department of Forensic Medicine, Wrocław Medical University, 50-367 Wroclaw, Poland; marta.rorat@gmail.com; 6 First Infectious Diseases Ward, Gromkowski Regional Specialist Hospital in Wrocław, 51-149 Wroclaw, Poland; 7 Department of Infectious Diseases and Neuroinfections, Medical University of Białystok, 15-089 Bialystok, Poland; piotr.czupryna@umb.edu.pl; 8 Pomeranian Center of Infectious Diseases, Department of Infectious Diseases, 80-210 Gdansk, Poland; lormar@gumed.edu.pl; 9 Department of Paediatrics and Infectious Diseases, Regional Hospital in Szczecin, 71-455 Szczecin, Poland; przciechanowski@spwsz.szczecin.pl; 10 Department of Infectious Diseases and Hepatology, Faculty of Medicine, Collegium Medicum in Bydgoszcz, Nicolaus Copernicus University, 87-100 Torun, Poland; d.kozielewicz@wsoz.pl (D.K.); m.pilarczyk@wsoz.pl (M.S.-P.); 11 Department of Infectious Diseases and Hepatology, Medical University of Łódź, 90-549 Lodz, Poland; annapiekar@gmail.com; 12 Department of Children’s Infectious Diseases, Medical University of Warsaw, 01-201 Warsaw, Poland; mpspiewak@gmail.com; 13 Department of Tropical Medicine and Epidemiology, Medical University of Gdańsk, 80-210 Gdansk, Poland; ksikorska@gumed.edu.pl; 14 Department of Infectious Diseases and Hepatology, Medical University of Lublin, 20-059 Lublin, Poland; magdalena.tudrujek@gmail.com; 15 Department of Infectious Diseases, Poznan University of Medical Sciences, 61-701 Poznan, Poland; bbolewska@ump.edu.pl; 16 7th Navy Hospital, 80-305 Gdansk, Poland; grzegorzangielski1@gmail.com; 17 Department of Adults’ Infectious Diseases, Medical University of Warsaw, 02-091 Warsaw, Poland; jdkowalska@gmail.com; 18 Regional Hospital of Infectious Diseases in Warsaw, 01-301 Warsaw, Poland; podlasin@zakazny.pl; 19 Clinical Department of Infectious Diseases in Chorzów, Medical University of Silesia, 41-500 Katowice, Poland; wlodek.maz@gmail.com; 20 Department of Infectious Diseases and Hepatology, Medical University of Silesia, 40-055 Katowice, Poland; bgrzesik@hoga.pl; 21 Department of Paediatrics and Infectious Diseases, Wroclaw Medical University, 50-367 Wroclaw, Poland; izabela.zaleska9@gmail.com; 22 Department of Infectious Diseases, Liver Diseases and Acquired Immune Deficiencies, Wroclaw Medical University, 51-149 Wrocław, Poland; ola.szymczak@gmail.com; 23 Department of Children’s Infectious Diseases, Provincial Jan Boży Hospital, 20-089 Lublin, Poland; franczak_paulina@wp.pl; 24 Department of Infectious Diseases and Allergology, Military Institute of Medicine, 04-141 Warsaw, Poland; kklos@wim.mil.pl; 25 Department of Infectious Diseases and Child Neurology, Poznan University of Medical Sciences, 60-572 Poznan, Poland; mfiglerowicz@gmail.com; 26 Department of Rheumatology, Rehabilitation and Internal Medicine, Poznan University of Medical Sciences, 61-701 Poznan, Poland; piotr_leszczynski@wp.pl; 27 2nd Department of Paediatrics, Centre of Postgraduate Medical Education, 02-507 Warsaw, Poland; izaqcharek@gmail.com; 28 General, Endocrine and Transplant Surgery Department, Medical University of Gdańsk, 80-214 Gdansk, Poland; hubert1grabowski@gmail.com

**Keywords:** epidemiology, SARS-CoV-2, clinical outcome, symptomatology, pandemic

## Abstract

Long-term analyses of demographical and clinical characteristics of COVID-19 patients can provide a better overview of the clinical course of the disease. They can also help understand whether changes in infection symptomatology, disease severity, and outcome occur over time. We aimed to analyze the demographics, early symptoms of infection, laboratory parameters, and clinical manifestation of COVID-19 patients hospitalized during the first 17 months of the pandemic in Poland (March 2020–June 2021). The patients’ demographical and clinical data (*n* = 5199) were extracted from the national SARSTer database encompassing 30 medical centers in Poland and statistically assessed. Patients aged 50–64 were most commonly hospitalized due to COVID-19 regardless of the pandemic period. There was no shift in the age of admitted patients and patients who died throughout the studied period. Men had higher C-reactive protein and interleukin-6 levels and required oxygenation and mechanical ventilation more often. No gender difference in fatality rate was seen, although the age of males who died was significantly lower. A share of patients with baseline SpO_2_ < 91%, presenting respiratory, systemic and gastrointestinal symptoms was higher in the later phase of a pandemic than in the first three months. Cough, dyspnea and fever were more often presented in men, while women had a higher frequency of anosmia, diarrhea, nausea and vomiting. This study shows some shifts in SARS-CoV-2 pathogenicity between March 2020 and July 2021 in the Polish cohort of hospitalized patients and documents various gender-differences in this regard. The results represent a reference point for further analyses conducted under the dominance of different SARS-CoV-2 variants.

## 1. Introduction

The outbreak of coronavirus disease (COVID-19) caused by severe acute respiratory syndrome coronavirus 2 (SARS-CoV-2) in late December 2019 in China quickly became an emerging, continuously evolving situation, spreading inevitably outside the Asian continent. It was declared a Public Health Emergency of International Concern at the end of January 2020 and a pandemic in March 2020 by the World Health Organization (WHO) [1]. Globally, nearly 84 million cases and 1.9 million deaths due to COVID-19 were reported by the end of 2020; both figures increased more than twofold in the following half-year. Although SARS-CoV-2 infections remain predominantly asymptomatic or mild, the clinical spectrum of COVID-19 is vast and includes severe progressive pneumonia and acute respiratory distress syndrome, both of which can be accompanied by cytokine storm, thrombosis, and multiple organ dysfunction [2,3]. The risk of severe COVID-19 is associated with increased age, obesity, male sex, and selected pre-existing medical conditions [4].

Since the publication of the first whole-genome sequence in January 2020, SARS-CoV-2 has been evolving, with numerous variants identified through genomic surveillance. In late 2020 and at the beginning of 2021, the emergence of variants posing higher public health risks, classified as variants of interest (VOIs) and variants of concern (VOCs), were observed. Two main evolutionary trajectories of SARS-CoV-2 include an increase in transmissibility (e.g., B.1.1.7 and B.1.617.2 variants) and evading host immune response (e.g., B.1.351 and others bearing E484K mutation) [5]. This has raised questions of whether these adaptive changes may be associated with increased vulnerability of different groups (e.g., younger, healthier subjects) to severe disease or influence the clinical presentation and outcome of COVID-19. It has been suggested that selected nonsynonymous mutations may be associated with more severe disease and inferior outcomes [6]. In vivo data indicated that infection with VOCs such as B.1.1.7 and B.1.351 reveal significant differences in pathogenicity with increased clinical progression and lower survival [7]. However, this has not been confirmed in the observational studies of hospitalized patients [8]. There has also been a discussion of whether shifts in the dominant SARS-CoV-2 variants in circulation may lead to changes in symptomatology [9].

Analyzing the long-term characteristics and trends of demographical and clinical data of patients hospitalized throughout a pandemic in a selected region can help assess whether there is a change in disease manifestation, severity and outcome, and understand the potential responsible factors. The present study summarized such data for COVID-19 patients hospitalized in 30 clinical centers in Poland between March 2020 and June 2021 and assessed whether there was any significant change in demographics (age, gender), early symptoms of infection, laboratory parameters, clinical manifestation, severity and outcome of the disease.

## 2. Materials and Methods

### 2.1. Data Collection

The data for this study was extracted from the SARSTer national database—an ongoing project led by the Polish Association of Epidemiologists and Infectiologists and supported by the Medical Research Agency (grant number 2020/ABM/COVID19/PTEILCHZ), collecting data on clinical characteristics of COVID-19 and treatment. Data for all COVID-19 patients hospitalized in 30 Polish centers between early March 2020 and mid-July 2021 were used in the analysis. Patients were diagnosed and treated with respect to applicable national recommendations for the management of COVID-19 [10,11,12,13].

The extracted demographical data included age, gender, body mass index (BMI) and comorbidities. Laboratory analysis data at admission included C-reactive protein (CRP), interleukin-6 (IL-6), procalcitonin (PCT), d-dimer, alanine aminotransferase (ALT), white blood cell count (WBC), absolute lymphocyte count (ALC), absolute neutrophil count (ANC) and platelet count (PLT). Early symptoms of infection before the treatment and oxygen saturation (SpO_2_) upon admission were also included. The clinical course of the disease was assessed with the ordinal scale based on the WHO recommendation, although modified to an 8-score version to fit the specificity of the Polish healthcare system and used in previous SARSTer studies [14,15]. The scores were given at baseline and after 7, 14, 21 and 28 days of hospitalization and were defined as follows: (1) not hospitalized, no activity restrictions; (2) not hospitalized, no activity restrictions and/or requiring oxygen supplementation at home; (3) hospitalized, does not require oxygen supplementation and does not require medical care; (4) hospitalized, requiring no oxygen supplementation, but requiring medical care; (5) hospitalized, requiring normal oxygen supplementation; (6) hospitalized, on non-invasive ventilation with high-flow oxygen equipment; (7) hospitalized, for invasive mechanical ventilation or extracorporeal membrane oxygenation; (8) death. Improvement in the clinical course of COVID-19 was defined as a reduction in the score of at least 2 points.

The demographical and clinical characteristics of patients were divided into five groups depending on the date of hospitalization: (i) early March 2020 to 30 June 2020, (ii) 1 July to 30 September 2020, (iii) 1 October to 31 December 2020, (iv) 1 January to 31 March 2021, and (v) 1 April to 15 July 2021. Two main pandemic phases were used for comparisons: early-phase (March to 30 September 2020) and late-phase (October 2020–July 2021). The former had a lower national number of identified infections (91,515), but shortages in equipment and medicine and a lower level of knowledge on COVID-19 among healthcare workers. The latter phase was characterized by high infection numbers (2,789,636) and an overwhelmed healthcare system, but the supplies of medicines (e.g., remdesivir) and oxygen, and experience in COVID-19 clinical course were much improved, while Polish recommendations of management of SARS-CoV-2 infections were already effectively implemented [10,11,12,13].

### 2.2. Statistical Analyses

The data analysis was done with Statistica v.13.1 (StatSoft, Tulsa, OK, USA). For continuous variables (age, BMI, length of hospitalization), differences were tested with a Student’s t test. For nominal categorical variables, differences in frequencies were tested with Pearson’s χ^2^ test. Trends in patient’s age and length of hospitalization were analyzed with a linear regression function and the coefficient of determination (R^2^). To evaluate associations between early symptoms of infection and the need for oxygen therapy, mechanical ventilation and death, the classical odds ratios (ORs) with a confidence interval were calculated according to the formulas given by Bland and Altman using MedCalc (MedCalc, Ostend, Belgium). To account for alpha inflation and limit the probability of type 1 error, Bonferroni corrections were applied in all multiple comparisons. A *p*-value < 0.05 was considered statistically significant.

## 3. Results

### 3.1. Demographic Characteristics

Overall, 5199 patients were included in this analysis, of whom 21.8% (*n* = 1133) were hospitalized between 6 March 2020 and 30 June 2020, 19.5% (*n* = 1012) between 1 July and 30 September 2020, 30.4% (*n* = 1581) between 1 October and 31 December 2020, 20.9% (*n* = 1087) between 1 January and 31 March 2021, and 7.4% (*n* = 386) between 1 April and 15 July 2021. Women constituted 45.7% of all patients; their share in considered periods fluctuated from 50.5% (till June 2020), 47.0% (July–September 2020), 42.0% (October–December 2020), 45.2% (January–March 2021) to 44.8% (April–July 2021). Fatality rates did not differ between women and men, although the age of male patients who died was significantly lower. The demographic breakdown of the studied population is presented in Table 1.

**Figure 1:**
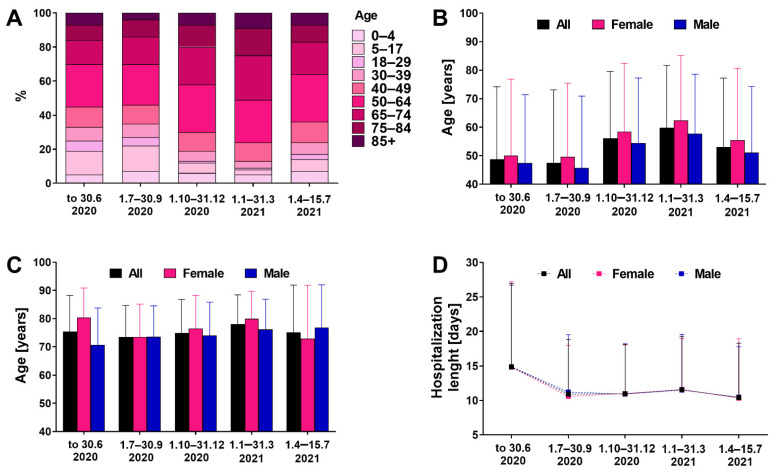
Structure of age (**A**) and mean ± SD age (**B**) of patients hospitalized in different periods of pandemic (*n* = 5199). (**C**) Age of patients who died in different periods of the pandemic. (**D**) Time of hospitalization (mean ± SD) in different periods of the pandemic.

The majority of hospitalized patients had at least one comorbidity and were aged ≥50 years (64.4%), with the highest share of individuals aged 50–64 (24–28%) regardless of the pandemic period (Figure 1A). There was no linear trend between patient’s age and month of hospitalization (y = 0.021x + 6.84; R^2^ = 0.018), also when analyzed separately for women (y = 0.022x + 6.56; R^2^ = 0.021) and men (y = 0.016x + 10.12; R^2^ = 0.0024). However, the age of hospitalized patients was lower in the early phase than the late phase of the pandemic (mean ± SD 48.2 ± 25.5 vs. 57.1 ± 23.1 years, *p* < 0.001) (Figure 1B).

The age of patients who died was similar across different periods (Figure 1C) and no linear trend was seen for the total population (y = 0.024x + 6.98; R^2^ = 0.0063), women (y = 0.0162x + 10.12; R^2^ = 0.0024) and men (y = 0.054x + 4.79; R^2^ = 0.034). The age of patients who died was similar in the early and late phases of the pandemic (mean ± SD 74.7 ± 12.2 vs. 76.3 ± 11.9 years, *p* > 0.05). The hospitalization length was the highest in March–June 2020 period (mean ± SD 14.9 ± 12.1 days) and then decreased to the 10–11 days range (Figure 1D). There was no linear trend between length and month of hospitalization for total population (y = 0.34x + 14.6; R^2^ = 0.021), group of women (y = 0.062x + 8.49; R^2^ = 0.021) and men (y = 0.063x + 8.86; R^2^ = 0.022). However, in general, the hospitalization stay was longer in the early pandemic phase compared to the late phase (mean ± SD 13.0 ± 10.5 vs. 11.1 ± 7.5 days).

### 3.2. Early Symptoms of Infection

**Figure 2:**
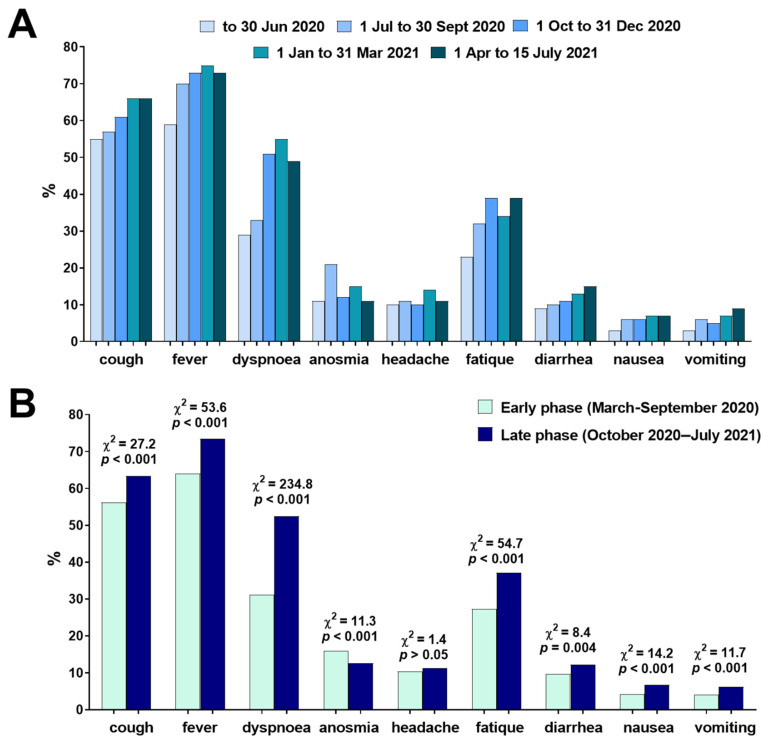
(**A**) Frequency of early COVID-19 symptoms presented by patients hospitalized in different periods of the pandemic (*n* = 5199) and (**B**) comparison in symptoms frequency between the early and late phase of the pandemic.

Fever (69.6%), cough (60.4%), and dyspnea (43.6%) were the most common early COVID-19 symptoms, followed by fatigue (33.0%), anosmia (13.9%), diarrhea (11.2%) and headaches (10.9%), while nausea (5.6%) and vomiting (5.3%) were the least commonly observed. Fluctuations in the frequency of early symptoms was observed in different periods of the pandemic. There was a steady increase of diarrhea reporting from 9.2% (March–June 2020) to 14.8% (April–July 2021). Compared to the early months of the pandemic, the frequencies of cough, fever, dyspnea and fatigue were also higher in subsequent months (Figure 2).

Significant gender differences in early symptoms were found. Compared to men, women had a lower frequency of cough (57.4 vs. 62.9%; χ^2^ = 16.3, *p* < 0.001), fever (64.0 vs. 74.4%; χ^2^ = 65.2, *p* < 0.001) and dyspnea (40.3 vs 46.4%; χ^2^ = 19.9, *p* < 0.001), but higher frequency of anosmia (15.3 vs. 12.8%; χ^2^ = 7.1, *p* = 0.007), headache (17.2 vs. 9.4%; χ^2^ = 20, *p* < 0.001), diarrhea (13.0 vs. 9.6%; χ^2^ = 15.1, *p* < 0.001), nausea (7.0 vs. 4.4%, χ^2^ = 17.5, *p* < 0.001) and vomiting (7.4 vs. 3.6%, χ^2^ = 36.0, *p* < 0.001). The presence of cough and dyspnea increased the odds of requiring oxygen therapy, mechanical ventilation and death in both women and men. Fever was associated with higher odds for oxygen therapy in women and men, and mechanical ventilation in men. Women presenting anosmia had lower odds for oxygen therapy and fatal outcome. Headache was associated with increased odds for oxygen therapy in women. Men and women presenting fatigue had higher odds for oxygen therapy. Gastrointestinal manifestations (diarrhea, nausea and vomiting) were not related to change in odds of the analyzed events (Table 2).

### 3.3. Laboratory and Clinical Characteristics

The summary of laboratory parameters at admission is provided in Table 3. Male patients were characterized by significantly higher inflammatory markers (CRP and IL-6), ALT, higher neutrophil count, and lower platelet count. Significant differences in the majority of considered parameters between patients hospitalized in the early and late phases of the COVID-19 pandemic were observed. The latter group had higher concentrations of CRP, IL-6, d-dimer and ALT, higher counts of WBC and neutrophils, but lower counts of lymphocytes and platelets (Table 3).

Considering that the odds for oxygen therapy and death were significantly lowered in subjects with anosmia (Table 2), the comparison of laboratory parameters between patients displaying or not displaying this symptom was performed. As shown, the former were characterized by significantly lower values of inflammatory markers: CRP (64.3 ± 70.7 mg/L vs. 71.2 ± 77.0 mg/L, *p* = 0.03), IL-6 (45.2 ± 76.5 pg/mL vs. 72.0 ± 187.8 pg/mL, *p* < 0.001) and PCT (0.2 ± 1.0 ng/mL vs. 0.5 ± 3.8 ng/mL, *p* < 0.001), and lower counts of WBC (6.4 ± 3.1 × 10^3^/µL vs. 7.1 ± 4.5 ×10^3^/µL) and neutrophils (4.5 ± 2.7 × 10^3^/µL vs. 4.9 ± 3.9 × 10^3^/µL).

**Figure 3:**
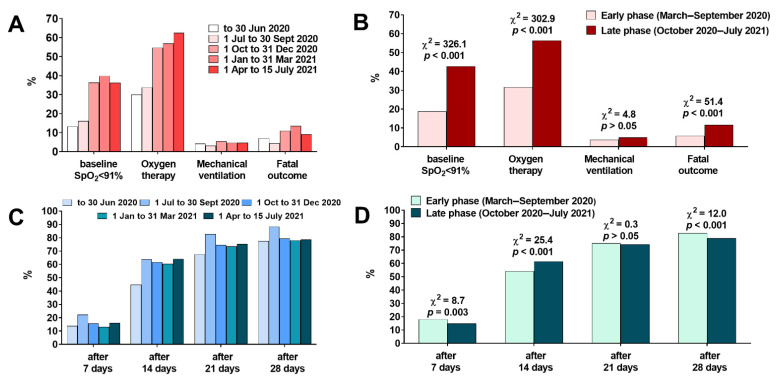
(**A**) The frequency of patients with baseline SpO_2_ < 91%, requiring oxygen therapy and mechanical ventilation, and with fatal outcome in different periods of the pandemic (*n* = 5199) and (**B**) comparison of these events between the early and late phase of the pandemic. (**C**) Percentage of patients with improvement in the clinical course of COVID-19 defined as a reduction in the score of at least 2 points on the ordinal 8-score scale (see Material & Methods for a detailed explanation of each score) in different periods of the pandemic and (**D**) comparison of these percentages between the early and late phase of the pandemic.

During the entire studied period, the share of patients with SpO_2_ < 91% at admission and requiring oxygen therapy was 32.5 and 46.1%, respectively, although their share increased since 1 October 2020 (Figure 3A). Compared to the late pandemic phase, the early phase of the pandemic had a significantly lower percentage of patients with SpO_2_ < 91% at admission requiring oxygen therapy. The overall percentage of patients requiring mechanical ventilation was 4.5%, with no difference between the early and late phases of the pandemic. The fatality rate in the studied period was 9.2% and increased significantly from 5.8% in the early pandemic phase to 11.6% in the late phase (Figure 3B). Men had higher odds for SpO_2_ < 91% (OR (95%CI) = 1.5 (1.3–1.6, *p* < 0.001), oxygen therapy (OR (95%CI) = 1.4 (1.3–1.6, *p* < 0.001) and mechanical ventilation (OR (95%CI) = 1.5 (1.2–2.0), *p* = 0.003), but not higher odds of death (OR (95%CI) = 1.1 (0.9–1.4, *p* > 0.05)). Clinical improvement, defined by a reduction in the score of at least 2 points on the ordinal scale, was less frequently recorded in the first period of the pandemic (March–July 2020), especially in the seven days and 14 days follow-ups (Figure 3C,D).

## 4. Discussion

This study provides a comprehensive overview of the COVID-19 patients hospitalized in Poland over the first 17 months of the pandemic and a reference point for further epidemiological analyses and comparisons. Consistent with various other observations, the investigated cohort was characterized mainly by elderly subjects suffering from at least one comorbidity, slightly more frequently represented by men.

The conducted analysis indicates some potential changes in pathogenicity of SARS-CoV-2 after September 2020, manifested by an increased share of patients with SpO_2_ < 91% and requiring oxygen therapy. The frequency of cough, fever, dyspnea also increased in later pandemic phases compared to the first three months. In general, the frequency of all considered symptoms except headache was higher in the late phase of the pandemic (October 2020–July 2021). Moreover, this phase was also characterized by patients exhibiting significantly increased levels of inflammatory markers, including IL-6, as well as differences in blood morphology: higher WBC and neutrophils counts but lower counts of lymphocytes and platelets. This may be due to the increase of the G superclade frequency in SARS-CoV-2 variants circulating in Poland in 2020 [16]. Its hallmark D614G substitution in spike protein was associated with increased transmissibility and higher viral loads [17]. Although the general human mortality was not found to be affected by D614G mutation, the animal studies demonstrated a modest increase in virulence—this slight increase may also be reflected in our observations [17,18].

According to some studies, infections with the B.1.1.7 (alpha) variant could be associated with higher mortality, although this was contradicted by other observations [18,19]. There have been some concerns, magnified by media reports, that the B.1.1.7 (alpha) variant may have a larger impact on the younger population by leading to a more severe clinical course of COVID-19 [20]. According to the national genomic surveillance, B.1.1.7 was steadily increasing in circulation in the Polish population since January 2021 to become dominant by mid-February and constitute over 80% of infections throughout March and April (>80% of infections). In this period, most hospitalizations were constituted by individuals >50 years, while a share of the younger population decreased between January and March 2021.

Furthermore, the age of patients who died did not differ throughout the considered 17 months. This observation contradicts various media reports, often based on the short-term experience of a single-center, claiming a gradual rise in COVID-19 deaths in younger individuals in the studied period. This is despite the worsening epidemiological situation in the late pandemic phase (October 2020–July 2021) in Poland compared to the early phase (March–September 2020) and the fact that COVID-19 vaccine rollout in the country was initiated on 27 December 2020 and 17.6 million people (46.5% of the population) had received at least one dose by 15 July 2021, while the elderly constituted a priority group in the national vaccination campaign. As shown previously, deaths from COVID-19 occurred very rarely in the fully vaccinated group and mostly concerned the immunocompromised, vaccine non-responders and individuals > 70 years with comorbidities [15].

At the same time, the length of hospital stay was the highest in the first months of the pandemic and decreased in the late pandemic phase. This should not be associated with any shifts in SARS-CoV-2 pathogenicity, as it was mostly due to epidemiological regulations enforced in Poland at the beginning of the pandemic (requirement to hospitalize patients for at least 14 days and obtaining two negative results for SARS-CoV-2 by PCR), as well as due to better experience in managing COVID-19 patients and increased availability of oxygen supply and treatment options. It is also likely that these aspects have also influenced the observed slight differences in the share of patients with clinical improvement rates between the early and late phases of the pandemic.

This study reports that gender differences in early COVID-19 symptoms were found. Respiratory symptoms (cough and dyspnea) and fever were more frequently observed in men, while women reported anosmia and gastrointestinal symptoms more often. This likely mirrors the differences in the immune response to the SARS-CoV-2 infection as indicated by significantly lower inflammatory markers (CRP and IL-6) in women. Previous research has shown that women reveal a more robust antiviral interferon response and increased adaptive immune response toward viral antigens, ultimately resulting in better viral control and lower disease severity [21]. Here, men also required oxygenation and mechanical ventilation more frequently, although it must be stressed that no gender disparity in fatality ratio was seen.

Although the previous research suggested that diarrhea may be related to worse COVID-19 outcomes, this was not seen in the present cohort [22]. The presence of gastrointestinal symptoms (diarrhea, nausea or vomiting) did not increase odds for oxygen therapy, mechanical ventilation and death, regardless of gender. In turn, patients with anosmia had lower odds for oxygen therapy and death. This is in line with previous findings linking smell loss with lower COVID-19 severity and better prognosis [23,24]. The mechanism behind these observations remains to be elucidated, although it could be hypothesized that the local inflammation of the olfactory bulb correlates with a more appropriate antiviral response. As shown in the present study, hospitalized subjects experiencing anosmia were characterized by significantly lower inflammatory markers at admission (IL-6, CRP and procalcitonin), confirming that the presence of this symptom is somewhat related to better control of the immune response to viral infection.

Although men were also characterized by higher mean levels of inflammatory markers (CRP and IL-6) and required oxygenation and mechanical ventilation more frequently, their hospitalization length and fatality rate were not increased compared to women. Male sex has been previously established as a risk factor for severe COVID-19 with the higher odds for death, as indicated by a meta-analysis of the global cases [21]. However, epidemiological reports from different U.S. states, Iran, Pakistan, and Finland, show no sex bias in mortality odds ratio [21]. The basis of these exceptional findings requires further research, although it may not solely be related to biological factors but also to socio-cultural and behavioral differences, as well as local healthcare capacities. In our study, women had comorbidities more often than men, and this may partially account for the lack of gender difference in survival.

It should be stressed that this study only included hospitalized COVID-19 patients. Thus, its observations, e.g., regarding changes in early symptoms of infection, may not necessarily translate to milder cases. Moreover, no genomic surveillance of SARS-CoV-2 was conducted for the studied cohort of patients; therefore, relationships between demographic and clinical characteristics and particular variants must be formulated with caution. However, the observations of this study do not translate to the B.1.617.2 (delta) variant that was first detected in Poland in May 2021 but became dominant in July 2021. Further studies are required to understand whether infections with B.1.617.2 are associated with different severity and outcomes in the Polish population.

## 5. Conclusions

In this study, we demonstrated shifts in SARS-CoV-2 pathogenicity that occurred between March 2020 and July 2021 in the Polish cohort of hospitalized patients and documented various gender differences in this regard. The clinical course of the disease did change, but it could have been caused, at least partially, by the varying burden on the health care system in different periods of the pandemic. This view is supported by the constant mean age of patients with a fatal outcome of the disease. The results represent a reference point for further analyses conducted under the dominance of different SARS-CoV-2 variants.

## Figures and Tables

**Table 1:** Demographic characteristics of COVID-19 patients hospitalized between 1 March 2020 and 15 July 2021, and differences in parameters between women and men measured with χ^2^ test or Student’s *t*-test.

	All (*n* = 5199)	Female (*n* = 2376)	Male (*n* = 2823)	*p*-Value
Age (years), mean ± SD (min–max)	53.4 ± 24.5 (0–100)	55.3 ± 25.4 (0–100)	51.9 ± 23.6 (0–97)	***p* < 0.001**
BMI (kg/m^2^), mean ± SD (min–max)	26.7 ± 6.4 (7.4–58.8)	26.1 ± 6.6 (7.4–56.9)	27.1 ± 6.3 (9.6–58.8)	***p* < 0.001**
Obese adults, % (*n*)	23.2 (1207)	22.4 (532)	23.9 (675)	*p* > 0.05
Comorbidities, % (*n*)	67.0 (3481)	68.6 (1629)	65.6 (1852)	***p* = 0.02**
Need for oxygenation, % (*n*)	44.9 (2333)	40.1 (952)	48.9 (1381)	***p* < 0.001**
Need for mechanical ventilation, % (*n*)	4.5 (233)	3.5 (84)	5.3 (149)	***p* = 0.003**
Time of hospitalization (days), mean ± SD	11.9 ± 8.9	11.9 ± 9.0	11.9 ± 8.8	*p* > 0.05
Fatality, % (*n*)	9.2 (479)	8.8 (208)	9.6 (271)	*p* > 0.05
Age of patients who died (years), mean ± SD (min–max)	75.9 ± 12.0	77.9 ± 11.7	74.3 ± 12.0	***p* < 0.001**

BMI: body mass index; COVID-19: coronavirus disease 2019. Statistically significant *p*-values are highlighted in bold.

**Table 2:** The odds ratio (95% confidence interval) for mechanical ventilation and death in relation to different early COVID-19 symptoms presented by hospitalized patients.

Symptom	Outcome	All (*n* = 5199)	Female (*n* = 2376)	Male (*n* = 2823)
Cough	Oxygen therapy	2.0 (1.7–2.2) ***p* < 0.001**	1.8 (1.5–2.1) ***p* < 0.001**	2.1 (1.8–2.4) ***p* < 0.001**
	Mechanical ventilation	1.9 (1.4–2.5) ***p* < 0.001**	2.2 (1.3–3.5) ***p* = 0.003**	1.0 (0.8–1.4) *p* > 0.05
	Death	0.8 (0.6–0.9) ***p* = 0.01**	0.8 (0.6–1.1) *p* > 0.05	0.7 (0.6–1.0) ***p* = 0.02**
Dyspnea	Oxygen therapy	6.3 (5.6–7.2) ***p*** **< 0.001**	5.3 (4.7–6.4) ***p*** **< 0.001**	7.2 (6.1–8.5) ***p* < 0.001**
	Mechanical ventilation	6.0 (4.3–8.3) ***p* < 0.001**	7.9 (4.4–14.1) ***p* < 0.001**	4.9 (3.3–7.4) ***p* < 0.001**
	Death	3.7 (3.0–4.5) ***p* < 0.001**	3.5 (2.6–4.7) ***p* < 0.001**	3.8 (2.9–5.1) ***p* < 0.001**
Fever	Oxygen therapy	2.0 (1.7–2.3) ***p* < 0.001**	1.7 (1.4–2.0) ***p* < 0.001**	2.2 (1.9–2.6) ***p* < 0.001**
	Mechanical ventilation	2.2 (1.6–3.2) ***p* < 0.001**	1.6 (1.0–2.6) *p* > 0.05	2.8 (1.7–4.7) ***p* < 0.001**
	Death	0.9 (0.7–1.1) *p* > 0.05	0.8 (0.6–1.1) *p* > 0.05	0.9 (0.7–1.2) *p* > 0.05
Anosmia	Oxygen therapy	0.8 (0.7–0.9) ***p* = 0.003**	0.7 (0.6–0.9) ***p* = 0.005**	0.9 (0.7–1.1) *p* > 0.05
	Mechanical ventilation	0.7 (0.5–1.1) *p* > 0.05	0.5 (0.2–1.1) *p* > 0.05	0.9 (0.5–1.5) *p* > 0.05
	Death	0.4 (0.3–0.6) ***p* < 0.001**	0.3 (0.2–0.6) ***p* < 0.001**	0.5 (0.3–0.8) ***p* = 0.004**
Headache	Oxygen therapy	0.7 (0.6–0.9) ***p* = 0.003**	0.6 (0.4–0.8) ***p* < 0.001**	0.9 (0.7–1.2) *p* > 0.05
	Mechanical ventilation	1.1 (0.7–1.6) *p* > 0.05	0.6 (0.3–1.4) *p* > 0.05	1.5 (0.9–2.5) *p* > 0.05
	Death	0.7 (0.5–1.0) *p* > 0.05	0.7 (0.4–1.2) *p* > 0.05	0.7 (0.4–1.1) *p* > 0.05
Fatigue	Oxygen therapy	1.6 (1.5–1.8) ***p* < 0.001**	1.4 (1.2–1.7) ***p* < 0.001**	1.9 (1.6–2.2) ***p* < 0.001**
	Mechanical ventilation	1.4 (1.1–1.8) ***p* = 0.02**	1.2 (0.7–1.8) *p* > 0.05	1.6 (1.1–2.2) ***p* = 0.007**
	Death	1.2 (0.9–1.5) *p* > 0.05	1.0 (0.8–1.4) *p* > 0.05	1.1 (0.7–2.0) *p* > 0.05
Diarrhea	Oxygen therapy	1.1 (0.9–1.3) *p* > 0.05	1.2 (0.9–1.5) *p* > 0.05	1.1 (0.9–1.5) *p* > 0.05
	Mechanical ventilation	1.1 (0.7–1.6) *p* > 0.05	1.0 (0.5–1.9) *p* > 0.05	1.1 (0.7–1.9) *p* > 0.05
	Death	1.1 (0.8–1.5) *p* > 0.05	1.0 (0.7–1.5) *p* > 0.05	1.2 (0.8–1.8) *p* > 0.05
Nausea	Oxygen therapy	1.0 (0.8–1.2) *p* > 0.05	1.0 (0.7–1.4) *p* > 0.05	1.0 (0.7–1.5) *p* > 0.05
	Mechanical ventilation	0.8 (0.4–1.5) *p* > 0.05	0.7 (0.2–1.8) *p* > 0.05	0.9 (0.4–2.2) *p* > 0.05
	Death	0.8 (0.5–1.3) *p* > 0.05	1.0 (0.6–1.8) *p* > 0.05	0.6 (0.3–1.2) *p* > 0.05
Vomiting	Oxygen therapy	0.9 (0.7–1.2) *p* > 0.05	1.0 (0.7–1.4) *p* > 0.05	0.9 (0.6–1.6) *p* > 0.05
	Mechanical ventilation	0.7 (0.4–1.4) *p* > 0.05	0.8 (0.3–2.0) *p* > 0.05	0.7 (0.3–2.0) *p* > 0.05
	Death	0.8 (0.5–1.2) *p* > 0.05	0.8 (0.4–1.4) *p* > 0.05	0.8 (0.4–1.7) *p* > 0.05

Statistically significant *p*-values are highlighted in bold.

**Table 3:** Laboratory parameters (mean ± SD) of hospitalized patients and differences between women and men, and early and late phase of the COVID-19 pandemic evaluated with Student’s *t*-test.

	All (*n* = 5199)	Female (*n* = 2376)	Male (*n* = 2823)	*p*-Value	Early Phase (*n* = 2145)	Late Phase (*n* = 3054)	*p*-Value
CRP, mg/L	70.2 ± 76.1	57.0 ± 68.9	81.3 ± 80.2	**<0.001**	50.4 ± 68.0	83.8 ± 78.5	**<0.001**
PCT, ng/mL	0.5 ± 3.5	0.4 ± 2.9	0.6 ± 3.9	>0.05	0.5 ± 4.7	0.5 ± 2.6	>0.05
IL-6, pg/mL	67.7 ± 175.2	58.9 ± 200.3	75.2 ± 150.3	**<0.001**	44.5 ± 150.1	80.1 ± 186.1	**<0.001**
d-dimer, ng/mL	1964.0 ± 6153.7	1865.5 ± 5309.3	2046.4 ± 6779.5	>0.05	1331.6 ± 4345.1	2361.7 ± 7029.2	**<0.001**
ALT, IU/L	40.6 ± 56.2	34.0 ± 50.1	46.2 ± 60.3	**<0.001**	34.9 ± 54.6	44.6 ± 56.9	**<0.001**
WBC, ×10^3^/µL	7.0 ± 4.4	6.7 ± 3.8	7.2 ± 4.7	**<0.001**	6.6 ± 4.2	7.3 ± 4.4	**<0.001**
Lymphocytes, ×10^3^/µL	1.4 ± 1.8	1.5 ± 1.4	1.4 ± 2.0	>0.05	1.6 ± 2.0	1.4 ± 1.6	**<0.001**
Neutrophils, ×10^3^/µL	4.9 ± 3.7	4.6 ± 3.2	5.1 ± 4.2	**<0.001**	4.2 ± 2.8	5.3 ± 4.2	**<0.001**
Platelets, ×10^3^/µL	227.2 ± 102.4	235.2 ± 98.7	220.5 ± 104.9	**0.003**	231.3 ± 98.0	224.8 ± 105.2	**0.04**

ALT: alanine aminotransferase; COVID-19: coronavirus disease 2019; CRP: C-reactive protein; IL-6: Interleukin-6; PCT: procalcitonin; SD: standard deviation; WBC: white blood cell. Statistically significant *p*-values are highlighted in bold.

## Data Availability

The datasets used and analyzed during the current study are available from the corresponding author upon reasonable request.
